# Genome-wide transcriptome analysis of hypothalamus in rats with inherited stress-induced arterial hypertension

**DOI:** 10.1186/s12863-015-0307-8

**Published:** 2016-01-27

**Authors:** Leonid O. Klimov, Nikita I. Ershov, Vadim M. Efimov, Arcady L. Markel, Olga E. Redina

**Affiliations:** Institute of Cytology and Genetics, Siberian Branch of Russian Academy of Sciences, Novosibirsk, Russian Federation; Novosibirsk State University, Novosibirsk, Russian Federation

**Keywords:** Stress-sensitive hypertension, Hypothalamus, Transcriptional profiling, RNA-Seq, ISIAH rats

## Abstract

**Background:**

The hypothalamus has an important role in the onset and maintenance of hypertension and stress responses. Rats with inherited stress-induced arterial hypertension (ISIAH), reproducing the human stress-sensitive hypertensive state with predominant involvement of the neuroendocrine hypothalamic-pituitary-adrenal and sympathoadrenal axes, were used for analysis of the hypothalamus transcriptome.

**Results:**

RNA-seq analysis revealed 139 genes differentially expressed in the hypothalami of hypertensive ISIAH and normotensive Wistar Albino Glaxo (WAG) rats. According to the annotation in databases, 18 of the differentially expressed genes (DEGs) were associated with arterial hypertension. The Gene Ontology (GO) functional annotation showed that these genes were related to different biological processes that may contribute to the hypertension development in the ISIAH rats. The most significantly affected processes were the following: regulation of hormone levels, immune system process, regulation of response to stimulus, blood circulation, response to stress, response to hormone stimulus, transport, metabolic processes, and endocrine system development. The most significantly affected metabolic pathways were those associated with the function of the immune system and cell adhesion molecules and the metabolism of retinol and arachidonic acid. Of the top 40 DEGs making the greatest contribution to the interstrain differences, there were 3 genes (*Ephx2, Cst3* and *Ltbp2*) associated with hypertension that were considered to be suitable for further studies as potential targets for the stress-sensitive hypertension therapy. Seven DEGs were found to be common between hypothalamic transcriptomes of ISIAH rats and Schlager mice with established neurogenic hypertension.

**Conclusions:**

The results of this study revealed multiple DEGs and possible mechanisms specifying the hypothalamic function in the hypertensive ISIAH rats. These results provide a basis for further investigation of the signalling mechanisms that affect hypothalamic output related to stress-sensitive hypertension development.

**Electronic supplementary material:**

The online version of this article (doi:10.1186/s12863-015-0307-8) contains supplementary material, which is available to authorized users.

## Background

Brain regulatory mechanisms play a major role in the control of sympathetic outflow and blood pressure (BP) regulation [1]. Based on the review of many studies, it was noted that most forms of hypertension are associated with a wide variety of functional changes in the hypothalamus [2]. In recent studies, progress has been made in understanding neurogenic aspects by the determination of global alterations in gene expression in key brain regions of animal models of neurogenic hypertension [3]. However, ongoing novel approaches and studies on different animal models are expected to lead to the identification of genes that serve as a common link between different forms of the disease [4] and a better understanding of the processes responsible for the increased sympathetic outflow and hypertension development in humans.

The aetiology of essential hypertension is multifactorial. As the studies on experimental animal models provide valuable information to elucidate the nature of polygenic traits [5], a number of animal models for essential hypertension are widely used. One of these is the ISIAH (Inherited Stress-Induced Arterial Hypertension) rat strain, which has been developed to study the mechanisms of stress-sensitive hypertension and its complications. The ISIAH rat strain was selected from outbred normotensive Wistar rats for an increased BP response to emotional stress caused by 30 min restraint in a cylindrical wire-mesh cell. The current population of ISIAH rats is characterized by elevation of both the basal arterial BP, which reaches up to 175.0 ± 3.5 mmHg in males and 165.0 ± 3.0 mmHg in females, and the stress-induced BP, which increases just after the mild emotional stress up to 195.0 ± 2.4 mmHg in males and 174.0 ± 3.2 mmHg in females [6, 7]. The high genetic homogeneity of the ISIAH strain was demonstrated by a DNA fingerprinting procedure using a multilocus microsatellite (CAC)_5_ probe [8]. Earlier studies showed that the ISIAH rats may be regarded as a model for the human stress sensitive hypertensive disease with predominant involvement of the neuroendocrine hypothalamic-pituitary-adrenal (HPA) and sympathoadrenal systems in the pathogenesis of the hypertensive state [9].

The recently developed next-generation sequencing technologies, widely used for transcriptome analysis (RNA-Seq approach), are making substantial contributions to the understanding of genome-wide expression and regulation [10]. In the current work, RNA-Seq technology was used for comparative analysis of the hypothalamic transcriptome in hypertensive ISIAH and inbred normotensive WAG rats. The goal of the study was to identify the differentially expressed genes (DEGs) and pathways involved in the differences of hypothalamic functions in the hypertensive ISIAH and normotensive WAG rats.

The study revealed multiple DEGs in the hypothalami of hypertensive ISIAH and normotensive WAG rats, including 18 DEGs known to be related to hypertension and the regulation of BP. These DEGs were associated with a diverse group of biological processes and pathways. Three DEGs were in the list of the top 40 DEGs defined as genes making the largest contribution to inter-strain differences. These DEGs may be considered potential candidates for further studies to better understand the mechanisms of hypertension development in ISIAH rats.

## Results

**Fig. 1:**
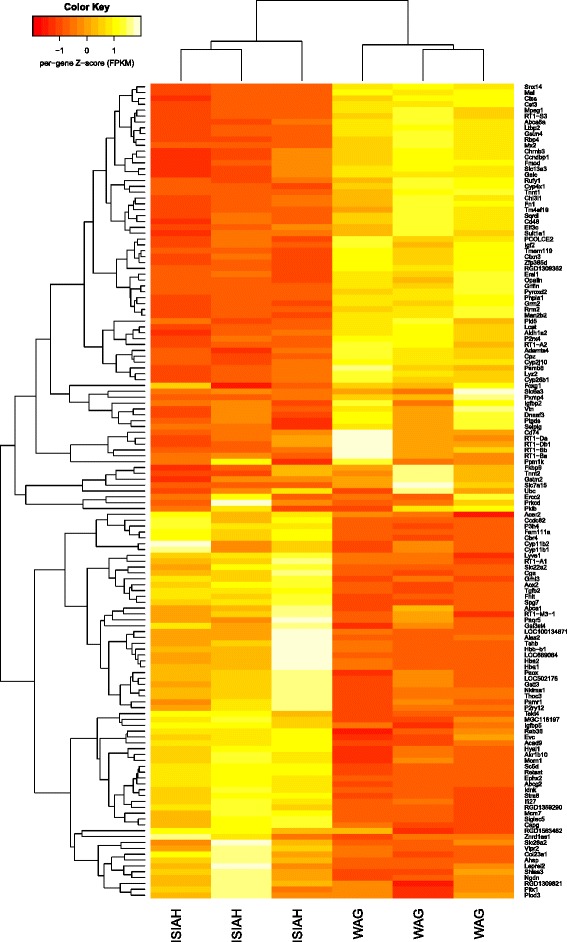
Heatmap of the differentially expressed genes in the hypothalami of the ISIAH and WAG rats. Dendrograms were constructed using hierarchical ‘complete linkage’ clustering by Euclidean distance. Yellow depicts genes that were up-regulated in a sample, and red depicts those that were down-regulated

Altogether, 11,369 annotated genes were defined as being expressed in the hypothalami of ISIAH and WAG rats and were used in comparative expression analysis, which revealed 139 DEGs. For a complete listing of these results, see Additional file 1. The hierarchical clustering using Euclidean distance is shown in Fig. 1. About half of the DEGs (71 genes, i.e., 51.1 %) were down-regulated in ISIAH rats. Two of the DEGs, *Ahsp* (alpha haemoglobin stabilizing protein, 1.9 FPKM) and *RGD1359290* (ribosomal_L22 domain containing protein, 46.5 FPKM), were detected in the hypothalamus of ISIAH rats but not in WAG rats.

### Functional annotation of DEGs

The most significant GO terms for biological processes, which may be related to the hypertensive state of ISIAH rats, and genes in related groups of DEGs are represented in Additional file 2. The major DEG groups were associated with the regulation of hormone levels, immune system processes, blood circulation, response to stimulus and regulation of the response to stimulus, transport, metabolic process, gland development, and regulation of the immune response. Some of these groups consisted of several subgroups describing the specificity of the processes. For instance, the group of DEGs associated with blood circulation included 5 genes that participate in the regulation of BP. The group labelled, ‘response to stimulus’, consisted of several subgroups based on the specificity of the stimuli. These subgroups predominantly consisted of ‘response to stress’, ‘response to hormone stimulus’, ‘response to inorganic substance’, and ‘response to external stimulus’. Many DEGs were associated with different metabolic processes including lipid metabolism, hormone metabolism, and vitamin A and catecholamine metabolic processes. These and some other DEG groups related to immune system processes, oxidation reduction, endocrine system development, cell activation and proliferation, indicated that there were multiple impairments in hypothalamic functions in hypertensive rats.

**Fig. 2:**
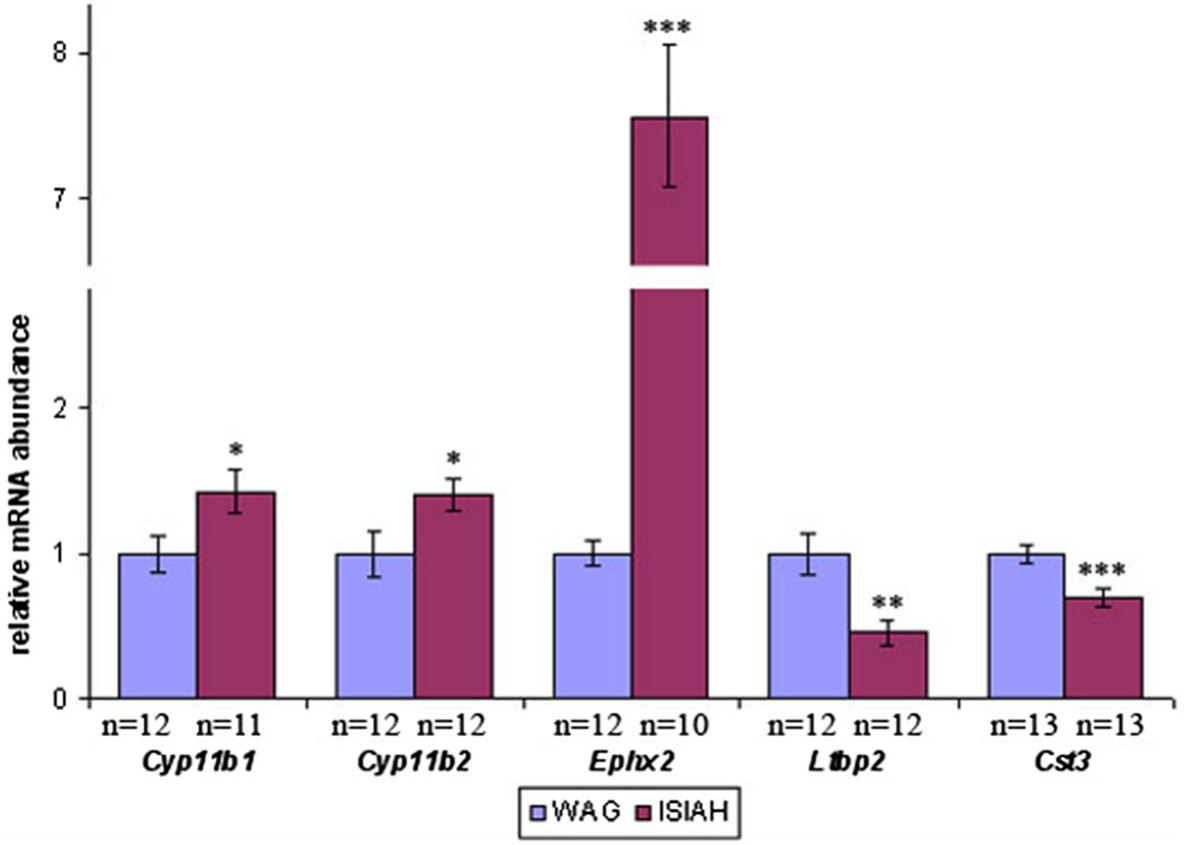
The relative mRNA abundance measured by qPCR. The relative mRNA abundance was calculated as the ratio of normalized (mRNA/Rpl30) mRNA level in experimental ISIAH samples to normalized (mRNA/Rpl30) mRNA level in control WAG samples. The normalized mRNA level in control samples of the WAG rats was assigned a value of 1. Vertical bars show the standard error of the mean, and significance of inter-strain difference is indicated by *p < 0.05, **p < 0.01, ***p < 0.001

Two DEGs known to be key players in corticosteroid biosynthesis and BP regulation (*Cyp11b1 and Cyp11b2*) were estimated to be up-regulated in the hypothalamus of hypertensive ISIAH rats. However, these genes were merged during the Cufflinks assembly and thus were tested as a single unit by the Cuffdiff program. To distinguish between their levels of expression, we performed quantitative real time polymerase chain reaction (qPCR). The results of qPCR confirmed the enhanced expression of both the *Cyp11b1* and *Cyp11b2* genes in the hypothalamus of ISIAH rats (Fig. 2).

The analysis in the Kyoto Encyclopedia of Genes and Genomes Pathway Database (KEGG) showed that the inter-strain differences in gene expression are related to six pathways that were significantly enriched (*p* < 0.05; Additional file 3). Four of these pathways were associated with the function of the immune system. All of the pathways (except for retinol metabolism) contained genes associated with hypertension and with central nervous system (CNS) diseases.

### Genes associated with hypertension and CNS diseases

Of the genes found to be differentially expressed in the hypothalami of ISIAH and WAG rats, 16 genes are annotated in the Rat Genome Database (RGD) as associated with hypertension, and two additional DEGs (*P2rx4,* and *Sult1a1*) were related to the regulation of BP according to the Database for Annotation, Visualization and Integrated Discovery (DAVID) tool. These 18 genes might be essential for hypertension development in ISIAH rats. Most of these genes are annotated in RGD as associated with diseases concomitant to hypertension, such as insulin resistance and CNS diseases (Table 1). Altogether, we found 26 DEGs referred to in RGD as associated with CNS diseases, including cerebrovascular disorders, brain infarction, and brain ischemia. Many of these genes were also related to immune system diseases (Table 2).

**Table 1:** Genes differentially expressed in ISIAH and WAG hypothalami and referred to in Databases as associated with hypertension and blood pressure regulation

Gene symbol	Acc.#	Gene name	log2 fold_change ISIAH/WAG
Rat Genome Database			
*Chi3l1* ^a,b^	NM_053560	chitinase 3-like 1 (cartilage glycoprotein-39)	−1.27
*Cst3* ^a,c,b^	NM_012837	cystatin C	−0.59
*Cyp11b1*	NM_012537	cytochrome P450, family 11, subfamily b, polypeptide 1	1.06
*Cyp11b2* ^a,c,b^	NM_012538	cytochrome P450, family 11, subfamily b, polypeptide 2	1.06
*Ephx2* ^a,c,b^	NM_022936	epoxide hydrolase 2, cytoplasmic	4.40
*Fn1* ^a^	NM_019143	no definition)	−0.98
*Gstm2*	NM_177426	glutathione S-transferase mu 2	−0.74
*Hyal1*	NM_207616	hyaluronoglucosaminidase 1	0.71
*Igfbp2* ^a,c^	NM_013122	insulin-like growth factor binding protein 2	−0.92
*Ltbp2*	NM_021586	latent transforming growth factor beta binding protein 2	−2.88
*Prkcd* ^a,c,b^	NM_133307	protein kinase C, delta	0.63
*Ptgds* ^a,c,b^	NM_013015	prostaglandin D2 synthase (brain)	−0.57
*RT1-Ba* ^a,b^	NM_001008831	RT1 class II, locus Ba	−0.80
*RT1-Bb* ^a,b^	NM_001004084	RT1 class II, locus Bb	−1.26
*RT1-Db1* ^a,b^	NM_001008884	RT1 class II, locus Db1	−0.76
*Slc6a3* ^a^	NM_012694	solute carrier family 6 (neurotransmitter transporter), member 3	−1.34
DAVID			
*P2rx4*	NM_031594	purinergic receptor P2X, ligand-gated ion channel 4	−0.86
*Sult1a1*	NM_031834	sulfotransferase family, cytosolic, 1A, phenol-preferring, member 1	−0.63

Genes associated with: ^a^- central nervous system diseases; ^b^- cerebrovascular disorders; ^c^- insulin resistance; ISIAH and WAG – rat strains used in the study. DAVID **-** Database for Annotation, Visualization and Integrated Discovery

**Table 2:** Genes differentially expressed in ISIAH and WAG hypothalami and referred to in Rat Genome Database as associated with central nervous system diseases

Gene symbol	Acc.#	Gene name	log2 fold_change ISIAH/WAG
*Abca1* ^a^	NM_178095	ATP-binding cassette, subfamily A (ABC1), member 1	0.65
*Abcg2*	NM_181381	ATP-binding cassette, subfamily G (WHITE), member 2	1.83
*Adamts4* ^a,b,c^	NM_023959	ADAM metallopeptidase with thrombospondin type 1 motif, 4	−0.59
*Chi3l1* ^a,b^	NM_053560	chitinase 3-like 1 (cartilage glycoprotein-39)	−1.27
*Cst3* ^a,b,c^	NM_012837	cystatin C	−0.59
*Ctss* ^a,b^	NM_017320	cathepsin S	−0.50
*Cyp11b2* ^b^	NM_012538	cytochrome P450, family 11, subfamily b, polypeptide 2	1.06
*Ephx2* ^b,d,c^	NM_022936	epoxide hydrolase 2, cytoplasmic	4.40
*Ercc2*	NM_001172809	excision repair cross-complementation group 2	2.30
*Fn1* ^a^	NM_019143	fibronectin 1	−0.98
*Galc*	NM_001005888	galactosylceramidase	−0.72
*Igf2* ^a,b,d,c^	NM_001190162	insulin-like growth factor 2 (Igf2), transcript variant 2	−1.09
*Igfbp2*	NM_013122	insulin-like growth factor binding protein 2	−0.92
*Mal*	NM_012798	mal, T-cell differentiation protein	−0.72
*P2ry12* ^a,b^	NM_022800	purinergic receptor P2Y, G-protein coupled, 12	0.59
*Prkcd* ^a,b,c^	NM_133307	protein kinase C, delta	0.63
*Ptgds* ^b^	NM_013015	prostaglandin D2 synthase (brain)	−0.57
*RGD1563482*	NM_001109065	similar to hypothetical protein FLJ38663	0.79
*RT1-Ba* ^a,b,d,c^	NM_001008831	RT1 class II, locus Ba	−0.80
*RT1-Bb* ^a,b^	NM_001004084	RT1 class II, locus Bb	−1.26
*RT1-Da* ^a^	NM_001008847	RT1 class II, locus Da	−0.63
*RT1-Db1* ^a,b^	NM_001008884	RT1 class II, locus Db1	−0.76
*Selplg* ^b^	NM_001013230	selectin P ligand	−0.78
*Slc6a3*	NM_012694	solute carrier family 6 (neurotransmitter transporter), member 3	−1.34
*Tgfb2* ^a^	NM_031131	transforming growth factor, beta 2	0.85
*Vtn* ^a^	NM_019156	vitronectin	−0.56

Genes associated with: ^a^- immune system diseases; ^b^- cerebrovascular disorders; ^c^- brain ischemia; ^d^- brain infarction; ISIAH and WAG – rat strains used in the study

### Transcription factors

Five transcription factor genes were differentially expressed in ISIAH and WAG hypothalami (Table 3). One of these (*Ercc2*) is annotated in RGD as being associated with hypertension. According to the GO functional annotation, two genes (*Ercc2* and *Grhl3)* were associated with response to stress, and three genes (*Foxg1, Mcm7*, and *Pitx1)* were related to metabolic processes. Moreover, *Pitx1* was related to the GO term ‘endocrine system development’ (Additional file 2).

**Table 3:** Transcription factor genes differentially expressed in ISIAH and WAG hypothalami

Gene symbol	Acc.#	Gene name	log2 fold_change ISIAH/WAG
*Ercc2*	NM_001172809	excision repair cross-complementation group 2	2.30
*Foxg1*	NM_012560	forkhead box G1	−0.86
*Grhl3*	NM_001106690	grainyhead-like 3 (Drosophila)	1.37
*Pitx1*	NM_053624	paired-like homeodomain 1	1.07
*Mcm7*	NM_001004203	minichromosome maintenance complex component 7	1.06

ISIAH and WAG – rat strains used in the study

### Genes making the greatest contribution to the inter-strain differences

The partial-least squares discriminant analysis (PLS-DA) was used to detect the genes that made the greatest contribution to the inter-strain differences. The PLS-DA Axes maximizing the distances between ISIAH and WAG rats were constructed (Additional file 4), and the correlation between gene expression and PLS-DA Axis 1 was defined. The genes that were designated as differentially expressed are shown in red in Figure in Additional file 5, and their polar position along the first PLS-DA Axis confirms their contribution to the existing inter-strain variations.

The top 40 DEGs that make the most significant contribution to the inter-strain differences possess different biological potencies (Table 4). According to RGD annotations, there were several genes related to CNS diseases (*Abcg2, Ephx2, RGD1563482, Tgfb2, Mal,* and *Cst3*), and 3 genes associated with hypertension (*Ephx2, Cst3,* and *Ltbp2*) are found on this list. These 3 genes may be considered to be potential candidates for further studies to better understand the mechanisms of hypertension development in ISIAH rats. Their differential transcription was validated by qPCR (Fig. 2). The comparison of the relative mRNA abundance is shown in Additional file 6. The correlation coefficient between the results from the two methods was 0.98.

**Table 4:** The top 40 differentially expressed genes making the greatest contribution to the inter-strain differences in hypothalamic function of ISIAH and WAG rats

Gene symbol	Acc.#	Correlation	log2 (fold_change) ISIAH/WAG	Definition	Function and/or process
*Retsat*	NM_145084	0.998	1.82	retinol saturase (all trans retinol 13,14 reductase)	oxidoreductase activity [64]
*Sc5d*	NM_053642	0.997	1.21	sterol-C5-desaturase	cholesterol biosynthetic process via lathosterol [65] fatty acid metabolic process; lathosterol oxidase activity [66]
*RGD1359290*	NM_001047898	0.996	was detected in ISIAH but not in WAG	Ribosomal_L22 domain containing protein RGD1359290	structural constituent of ribosome
*Abcg2* ^a^	NM_181381	0.994	1.83	ATP-binding cassette, subfamily G (WHITE), member 2	transmembrane transport [67]
*Ephx2* ^a,b^	NM_022936	0.993	4.40	epoxide hydrolase 2, cytoplasmic	positive regulation of blood pressure [24]
*Fam111a*	NM_001109163	0.993	5.12	family with sequence similarity 111, member A	DNA replication [68]
*RGD1563482* ^a^	NM_001109065	0.990	0.79	similar to hypothetical protein FLJ38663	aminoacyl-tRNA hydrolase activity
*Stra6*	NM_001029924	0.990	0.70	stimulated by retinoic acid 6	transports retinol across the cell membrane [18]
*Aox2*	NM_001008522	0.985	1.47	aldehyde oxidase 2	oxidoreductase activity
*Fhit*	NM_021774	0.983	1.46	fragile histidine triad	nucleotide metabolic process [69]
*Tekt4*	NM_001013965	0.981	1.00	tektin 4	protein binding [70]
*LOC689064*	NM_001111269	0.980	3.23	beta-globin	oxygen transporter activity
*Grhl3*	NM_001106690	0.976	1.37	grainyhead-like 3 (Drosophila)	may stimulate migration of endothelial cells [71]
*Acad9*	NM_181768	0.975	0.59	acyl-CoA dehydrogenase family, member 9	oxidoreductase activity catalyze the rate-limiting step in the beta-oxidation of fatty acyl-CoA
*Tgfb2* ^a^	NM_031131	0.975	0.85	transforming growth factor, beta 2	promotes the survival of dopaminergic neurons [37]
*Spg7*	NM_181388	0.975	0.58	spastic paraplegia 7 homolog (human)	metalloendopeptidase activity [72] nervous system development [73]
*Idnk*	NM_001037362	0.974	0.91	idnK, gluconokinase homolog (E, coli)	gluconokinase activity
*Lyz2*	NM_012771	−0.968	−0.68	lysozyme 2	lysozyme activity
*Mal* ^a^	NM_012798	−0.971	−0.72	mal, T-cell differentiation protein	myelination [74]; structural constituent of myelin sheath [75]
*Lcat*	NM_017024	−0.972	−0.77	lecithin cholesterol acyltransferase	phospholipase A2 activity [76] cholesterol metabolic process [77]
*PCOLCE2*	NM_001127640	−0.972	−2.12	procollagen C-endopeptidase enhancer 2	collagen binding [78]
*RT1-S3*	NM_001008886	−0.974	−2.03	RT1 class Ib, locus S3	antigen processing and presentation of peptide antigen via MHC class I
*Cst3* ^a,b^	NM_012837	−0.976	−0.59	cystatin C	prevents oxidative stress-induced death [44]
*Cyp2j10*	NM_001134980	−0.977	−0.94	cytochrome P450, family 2, subfamily j, polypeptide 10	EETs biosynthesis [79]
*Gstm4*	NM_001024304	−0.978	−1.75	glutathione S-transferase mu 4	glutathione binding glutathione metabolic process
*Grm2*	NM_001105711	−0.979	−1.81	glutamate receptor, metabotropic 2	regulation of synaptic transmission, glutamatergic [80]
*Mx2*	NM_134350	−0.979	−1.29	myxovirus (influenza virus) resistance 2	innate immune response [81]
*Zfp385d*	NM_001013992	−0.980	−0.74	zinc finger protein 385D	zinc ion binding
*Tnnt1*	NM_001277260	−0.981	−4.37	troponin T type 1 (skeletal, slow)	regulation of muscle contraction [82]
*Cpz*	NM_031766	−0.982	−0.89	carboxypeptidase Z	zinc ion binding metallocarboxypeptidase activity extracellular matrix homeostasis
*Pyroxd2*	NM_001004261	−0.983	−2.86	pyridine nucleotide-disulphide oxidoreductase domain 2	oxidoreductase activity
*Rbp4*	NM_013162	−0.985	−1.49	retinol binding protein 4, plasma	retinol transporter activity [83]
*Abca8a*	NM_001281824	−0.987	−0.65	ATP-binding cassette, subfamily A (ABC1), member 8a	transporter activity lipid transport transmembrane transport
*Mpeg1*	NM_022617	−0.989	−2.31	macrophage expressed 1	
*Grifin*	NM_057187	−0.989	−4.44	galectin-related inter-fiber protein	carbohydrate binding
*Ltbp2* ^b^	NM_021586	−0.989	−2.88	latent transforming growth factor beta binding protein 2	decreases fibroblast adhesion to fibronectin [84] negatively regulates coalescence of oxytalan fibers induced by stretching stress [85]
*Snx14*	NM_001108174	−0.990	−0.85	sorting nexin 14	phosphatidylinositol-3,5-bisphosphate binding [86]
*Pnpla1*	NM_001191841	−0.993	−2.59	patatin-like phospholipase domain containing 1	lipid catabolic process
*RT1-A2*	NM_001008829	−0.993	−1.98	RT1 class Ia, locus A2	antigen processing and presentation of peptide antigen via MHC class I
*RGD1309362*	NM_001024884	−0.995	−2.46	similar to interferon-inducible GTPase	

Genes associated with: ^a^central nervous system diseases; ^b^-hypertension; ISIAH and WAG – rat strains used in the study

## Discussion

Mapping and quantifying mammalian transcriptomes by deep sequencing (RNA-Seq) provides a digital measure of the presence and prevalence of transcripts [11]. In the current study, an RNA-Seq approach was performed to detect genes with altered transcriptional activity in the hypothalamus of hypertensive ISIAH rats compared to normotensive WAG rats. However, only 18 out of the 139 DEGs found in the current study were characterized as being associated with the hypertension. This could be explained by genetic drift during the selection process, which could result in randomly fixed alleles contributing to differences in gene expression but not related to disease itself. The functional annotation of DEGs helped to define the main biological processes that might contribute to inter-strain differences. This analysis showed that almost all GO groups contained the DEGs associated with hypertension (see Additional file 2). These results highlight once again that many genes and biological processes may contribute to the stress-sensitive hypertension development.

As expected, GO annotation revealed multiple DEGs related to the hormonal nature of hypothalamic function. The group of eight DEGs participating in the regulation of hormone levels (see Additional file 2) might play an important role in the orchestration of the changes in physiological and metabolic processes in ISIAH hypothalamus. This group contained several genes associated with hypertension. Two genes in this group, cytochrome P450, family 11, subfamily b, polypeptide 1 (*Cyp11b1*) and cytochrome P450, family 11, subfamily b, polypeptide 2 (*Cyp11b2*), are known to be key players in steroidogenic pathways [12]. These genes are particularly known to be involved in corticosterone and aldosterone biosynthesis and are widely known to be regulators of BP. It was shown that the small amounts of aldosterone synthesized in the brain of Dahl salt-sensitive hypertensive rats could provide a local ligand for autocrine or paracrine activation of the mineralocorticoid receptor [13], and this was important in the genesis of salt-sensitive hypertension [14]. Aldosterone synthesized in the brain contributes to salt-sensitive hypertension through an increased salt appetite, sympathetic drive and vasopressin release [15]. The up-regulation of the *Cyp11b2* gene found in the current study may result in aldosterone excess in the hypothalamus of ISIAH rats and may also contribute to the development of stress-sensitive hypertension.

Several other DEGs participated in the regulation of hormone levels were related to retinol metabolism: aldehyde dehydrogenase 1 family, member A2 (*Aldh1a2*), cytochrome P450, family 26, subfamily b, polypeptide 1 (*Cyp26b1*), retinol binding protein 4, plasma (*Rbp4*), and retinol saturase (*Retsat*). Retinoic acid (RA) is an essential signalling molecule. In the adult brain, RA has been shown to be involved in synaptic plasticity, the induction of neural differentiation, motor axon outgrowth and nerve regeneration [16, 17]. In the current study, *Rbp4* and *RetSat* were in a list of the top 40 DEGs that contribute the most to inter-strain differences (Table 4). Additionally, in this list, there was one additional gene, stimulated by retinoic acid 6 (*Stra6*), related to retinol transport across the cell membrane [18]. These data suggest that retinol metabolism is likely to make a substantial contribution to inter-strain differences in hypothalamic functions; however, the direct influence of this pathway to BP regulation has not been defined so far.

One additional gene associated with the regulation of hormone level was *Cga* (glycoprotein hormones, alpha polypeptide). It encodes the alpha subunit of several glycoprotein hormones: chorionic gonadotropin, luteinizing hormone, follicle stimulating hormone, and thyroid stimulating hormone. Its up-regulation in the ISIAH hypothalamus may contribute to a variety of biological processes related to endocrine system functioning in hypertensive rats.

The *Sult1a1* (sulfotransferase family, cytosolic, 1A, phenol-preferring, member 1) gene was associated with several biological processes including the regulation of hormone levels, the regulation of BP, and the response to hormone stimulus (Additional file 2). Rat sulfotransferase Sult1a1 catalyzes the sulfation of neurotransmitters and xenobiotic phenolic compounds [19]. Sulfation usually leads to the inactivation of biological signalling molecules including catecholamines [20]. It was shown that the experimental reduction of norepinephrine content in the anterior hypothalamus induced an increase in arterial blood pressure and heart rate [21]. As Sult1a1 sulfates catecholamines, the down-regulation of this gene in the ISIAH hypothalamus may be adaptive and directed against the excessive elevation of BP.

Two other genes related to the regulation of the BP level that are differentially expressed in ISIAH and WAG hypothalami were the purinergic receptor P2X, the ligand-gated ion channel 4 (*P2rx4*) and epoxide hydrolase 2, cytoplasmic (*Ephx2*). The product of the *P2rx4* gene belongs to the family of purinoceptors for adenosine triphosphate. This receptor functions as a ligand-gated cation-selective channel. Endothelial P2X4 channels regulate BP and vascular remodelling. P2rx4 has a key role in the response of endothelial cells to changes in blood flow. P2rx4(−/−) mice have higher BP levels and excrete smaller amounts of NO products in their urine than wild-type mice [22]. Based on this, we suggest that down-regulation of the *P2rx4* gene in the ISIAH hypothalamus might contribute to hypertension development.

*Ephx2* encodes the soluble epoxide hydrolase (sEH) that metabolizes the epoxyeicosatrienoic acids (EETs). EETs modulate ion transport and gene expression and produce vasorelaxation, as well as anti-inflammatory and pro-fibrinolytic effects [23]. sEH inhibition lowers arterial BP in angiotensin II-induced hypertension [24]. sEH overexpression was linked to hypertension in the spontaneously hypertensive rats [25]. *Ephx2* was also considered to be one of the gatekeeper genes that contributes to programmed hypertension [4]. In our study, *Ephx2* was one of several DEGs related to arachidonic acid (AA) metabolism. The two other DEGs, cytochrome P450, family 2, subfamily j, polypeptide 10 (*Cyp2j10*) and cytochrome P450, family 4, subfamily x, polypeptide 1 (*Cyp4x1*)*,* are also known to encode enzymes that regulate EET production. The down-regulation of these two genes and the enhanced expression of the *Ephx2* gene in the ISIAH hypothalamus may lead to a reduced amount of EETs and contribute to hypertension development in ISIAH rats. In the current study, *Ephx2* and *Cyp2j10* were in a list of the top 40 DEGs that contribute the most to inter-strain differences (Table 4).

One more DEG related to AA metabolism was prostaglandin D2 synthase (brain) (*Ptgds*). The decreased expression of *Ptgds* found in the current study may lead to a reduced amount of prostaglandin D2, which is a major brain prostaglandin that functions as a neuromodulator and/or trophic factor in the CNS [26]. It is involved in smooth muscle contraction/relaxation and is a potent inhibitor of platelet aggregation [27].

Both GO annotation and KEGG analysis revealed highly significant differences in the expression of genes belonging to immune system processes in the hypothalami of ISIAH and WAG rats. It is long known that immune system changes play a role in hypertension and that extensive bidirectional interactions between the sympathetic nervous system and the immune system exist [28, 29]. Recent studies have shown that both innate and adaptive immunity contribute to hypertension [30]. Major histocompatibility complex (MHC) class I molecules are ligands for the killer-cell immunoglobulin-like receptors that are expressed by natural killer (NK) cells and T cells. The interactions between these molecules contribute to both innate and adaptive immunity [31]. MHC class-II molecules are key participants in immune activation events in autoimmunity [32]. It was shown that mice lacking adaptive immune cells, including recombinase-activating gene-deficient mice and rats and mice with severe combined immunodeficiency, have blunted hypertension responses to stimuli such as ANG II, high salt, and norepinephrine [33]. MHC molecules are rare in healthy brain tissue, but are heavily expressed on microglial cells after inflammatory or neurodegenerative processes [34]. The transcription of most of the DEGs that encode MHC molecules was decreased in the hypothalamus of ISIAH rats. However, two genes, *RT1-A1* (RT1 class Ia, locus A1) and *RT1-M3-1* (RT1 class Ib, locus M3, gene 1), were up-regulated. Earlier, it was shown that the expression of the RT1-A1 molecule protects targets from NK lysis [35]. In another study it was found that the expression of RT1-A1 became up-regulated in glial cells after mechanical nerve injury [36]. We can suppose that the elevated transcription of *RT1-A1* in the ISIAH hypothalamus may play a protective role.

Altogether, there were three genes from the list of the top 40 DEGs, RT1 class Ia, locus A2 (*RT1-A2*), RT1 class Ib, locus S3 (*RT1-S3*), and transforming growth factor, beta 2 (*Tgfb2*), that made the most significant contribution to the inter-strain differences in the group ‘immune system process’ (Table 4). The possible role of the MHC molecules in pathology development was discussed above. TGFb2 is known to promote the survival of dopaminergic neurons [37]. The hypothalamic changes in neurotransmitter and neuromodulator functions via the sympathetic nervous system appear to play a predominant role in most forms of hypertension [2]. *Tgfb2* was one of several DEGs identified in the current study that were related to endocrine system development (see Additional file 2). Two other genes related to this group and not discussed above were solute carrier family 6 (neurotransmitter transporter), member 3 (*Slc6a3*) and paired-like homeodomain 1 (*Pitx1*). The *Slc6a3* gene encodes the dopamine transporter. Its primary physiological role is the regulation of extracellular dopamine by means of rapid transport (reuptake) of dopamine back into the dopaminergic presynaptic terminals. Increased expression of the dopamine transporter leads to a loss of dopamine neurons and oxidative stress [38]. Therefore, the up-regulation of *Tgfb2* and the decreased expression of *Slc6a3* found in the hypothalamus of ISIAH rats might have a protective role for dopaminergic neurons.

*Pitx1* encodes a homeobox transcription factor involved in transcriptional activation of the pro-opiomelanocortin (*Pomc*) gene [39, 40]. The increased hypothalamic POMC presumably attenuates weight gain [41], activates sympathetic neurotransmission to adipose tissues, accelerates energy expenditure [42], and could be, at least in part, responsible for the increased sensitivity to insulin [43]. However, *Pomc* transcription was not significantly increased in the ISIAH hypothalamus in our study. Therefore, the up-regulation of *Pitx1*, may be essential for the other signalling pathways in the hypothalamus of hypertensive rats.

The hypothalamus is known as a brain region involved in the regulation of body homeostasis and stress responses. In the current study, multiple DEGs were associated with the response to different stimuli such as stress, hormonal stimulus, inorganic substance, and external stimuli (Additional file 2). As the study was conducted on rats not exposed to the stress, the DEGs in the group ‘response to stress’ might be essential for stress-sensitive hypertension development. Altogether, we found nine genes (*Cst3, Cyp11b2, Ephx2, Fn1, Igfbp2, P2rx4, Prkcd, RT1-Ba,* and *Sult1a1*) annotated in the RGD as associated with hypertension in this group. Two of these genes cystatin C (*Cst3*) and epoxide hydrolase 2, cytoplasmic (*Ephx2*) were also present in the list of top 40 DEGs defined as making the largest contribution to inter-strain differences (Table 4). Cystatin C (*Cst3* gene) is an inhibitor of cysteine proteinases. Expression of cystatin C has protective effects against various oxidative stresses that induce cell death [44]. The studies of hypothalamic mechanisms underlying the neurogenic hypertension in mice showed that inflammation and oxidative stress via reactive oxygen species generation could contribute to oxidative damage and activation of the sympathetic nervous system leading to an increase in BP [45]. Therefore, the decreased transcription of the *Cst3* gene in the ISIAH hypothalamus may contribute to hypertension development as well as excessive transcription of the *Ephx2* gene discussed above.

Altogether, the group ‘response to stimulus’ contained 13 genes (*Abcg2, Cst3, Ephx2, Grhl3, Grm2, Gstm4, Lcat, Lyz2, Mx2, Rbp4, RT1-A2, RT1-S3,* and *Tgfb2*) included in the list of top 40 DEGs that contribute the most to inter-strain differences in hypothalamic function in the current study. The known activities of genes described in Table 4 or discussed above underscore the diversity of the mechanisms that contribute to the inter-strain differences. These genes might be useful in further studies to elucidate the mechanisms accompanying the stress-sensitive hypertension development.

In the current study, only 3 genes associated with hypertension (*Ephx2, Cst3* and *Ltbp2*) appeared on the list of the top 40 DEGs that make the largest contribution to inter-strain differences. We suggest that these 3 genes might be of interest for their further studies as potential therapeutic targets for stress-sensitive hypertension therapy. One of these genes, *Ephx2,* was already proposed as a target for the treatment of hypertension [46]. *Ltbp2* had previously been proposed to be a potential biomarker for atrial stress [47] and was patented as a biomarker for cardiovascular, hematological, neurological, endocrinological, and urological diseases, as well as cancer [48]. As discussed above, cystatin C has protective effects against various oxidative stresses. Therefore, its contribution to BP regulation may be expected.

The analysis of different animal models of hypertension helps to identify the genes which may serve as a common link between different forms of the disease [4]. In the current study, we found several common genes with changed transcriptional activity in the hypothalami of hypertensive ISIAH rats and hypertensive Schlager mice that represent a genetic model of neurogenic hypertension (Table 5) [45]. One of these genes, chitinase 3-like 1 (cartilage glycoprotein-39) (*Chi3l1*), is known to be associated with hypertension. The protein encoded by this gene is thought to play a role in the process of inflammation [49], tissue remodelling [50] and reactive oxygen species production [51]. The other genes listed in Table 5 were not previously related to the regulation of blood pressure, but, as they are common between two models of the disease, these genes may be considered promising targets for further studies directed to understanding the processes contributing to increased sympathetic outflow and hypertension development. During the last decade, the results of high-throughput biological experiments made a significant contribution to understanding the molecular bases of complex diseases. RNA-Seq is one of the recently developed approaches to transcriptome profiling which provides a far more precise measurement of levels of transcripts than other methods [52]. However, it has some limitations. The main limitation of RNA-Seq analysis is its expensiveness. The other limitation is the relative impossibility of technical validation of all the multiple DEGs found in the experiment. Therefore, the current study was conducted with the use of only three biological replicates in both experimental and control groups, and the expression of only several DEGs was validated technically. However, it was shown earlier that the results from RNA-Seq analysis showed high levels of reproducibility for both technical and biological replicates [53, 54]. In the current study, we found a strong correlation (*r* = 0.98) between the qPCR and RNA-Seq data. These results are similar to those reported by other groups [53].

**Table 5:** The list of the common genes with changed transcriptional activity in hypothalami of hypertensive ISIAH rats and hypertensive Schlager mice (26 weeks old), which represent a genetic model of neurogenic hypertension

Gene symbol	Acc.#	Gene definition	log2 (fold_change) ISIAH/WAG	Fold difference in Schlager mice^b^
*Acer2*	NM_001107943	alkaline ceramidase 2	0.60	1.69
*Chi3l1* ^a^	NM_053560	chitinase 3-like 1 (cartilage glycoprotein-39)	−1.27	−1.73
*Fhit*	NM_021774	fragile histidine triad	1.46	−1.3
*Galc*	NM_001005888	galactosylceramidase	−0.72	−1.42
*Nkiras1*	NM_001107252	NFKB inhibitor interacting Ras-like 1	0.59	1.19
*Paqr5*	NM_001014092	progestin and adipoQ receptor family member V	0.89	1.25
*Tnnt1*	NM_001277260	troponin T type 1 (skeletal, slow)	−4.37	−2.01

^a^- Genes associated with hypertension; ^b^Values represent mean of the fold difference between hypertensive and normotensive samples. Positive fold difference values indicate higher expression in hypertension and negative fold difference values indicate higher expression in normotension [45]

## Conclusion

The results of comparative transcriptional profiling of the hypothalamus in hypertensive ISIAH and normotensive WAG rats helped to identify multiple DEGs, including 18 genes associated with hypertension and regulation of BP. These DEGs were related to the diversity of biological processes and pathways contributing significantly to the inter-strain differences related to hypothalamic function. The discussion demonstrated that many genes found to be differentially expressed in ISIAH and WAG hypothalami might be responsible for pathology development, and that the changed expression of other genes may exert compensatory effects and be directed toward the restoration of homeostasis. As the number of hypertensive genes was considerable, we were not able to discuss all of them in details. Moreover, the discussion of some genes was complicated by uncertainty about their roles, especially within the hypothalamus. In the current study, we defined 3 genes associated with hypertension (*Ephx2, Cst3* and *Ltbp2*) that might be of interest for further studies as potential therapeutic targets for stress-sensitive hypertension therapies. Our findings provide a basis for identification of potential biomarkers of stress-sensitive hypertension and for further investigation of the signalling mechanisms that affect hypothalamic output related to hypertension development and the resulting sympathetic nervous system effects on other tissues and the organism as a whole.

## Methods

### Animals

Two inbred rat strains were used in the current study. Both hypertensive ISIAH (Inherited Stress Induced Arterial Hypertension) and normotensive Wistar Albino Glaxo (WAG/GSto-Icgn) rats were bred in the Center for Genetic Resources of Laboratory Animals at the Institute of Cytology and Genetics, Siberian Branch of the Russian Academy of Sciences (Novosibirsk, Russia, RFMEFI61914X0005 and RFMEFI61914X0010). All rats were maintained under standard conditions with free access to food and water. One week before the systolic arterial BP measurement, animals were placed in individual cages. The systolic arterial BP was measured indirectly by the tail-cuff method under short-term ether anaesthesia to exclude the effect of psychological stress induced by the measuring procedure. In RNA-seq experiments, the 3-month-old ISIAH (*n* = 3) and WAG (*n* = 3) males were used. Their systolic arterial BP was 171.7 ± 1.22 mmHg in ISIAH and 116.33 ± 1.86 mmHg in WAG males. The rats were decapitated 6 days after BP measurement, and their hypothalami were isolated according to known anatomical location [55]. All samples were stored in RNALater (Qiagen, Chatsworth, CA) at −70 °C until use. All animal experiments were approved by the Institute’s Animal Care and Use Committee.

### RNA-Seq analysis

The collected samples were sent to JSC Genoanalytica (Moscow, Russia) where mRNA was extracted using Dynabeads mRNA Purification Kit (Ambion, USA). cDNA libraries were constructed using NEBNext mRNA Library Prep Reagent Set for Illumina (NEB, USA) following the manufacturer’s protocol and subjected to single-end sequencing on Illumina HiSeq1500 with read length of 50 bases. Three hypothalami from ISIAH and three hypothalami from WAG rats were run as experimental replicates. The resulting fastq-formatted sequencing data were mapped to the RGSC Rnor_5.0\rn5 reference genome using Tophat2 aligner [56] and ENSEMBL/RefSeq gene annotation. A quality assessment of the mapped data was performed using the module ‘CollectRnaSeqMetrics’ from Picard tools suite (http://broadinstitute.github.io/picard/). The summary statistics for each sequenced library is given in Additional file 7. The Cufflinks/Cuffdiff programs were then used to estimate gene expression levels in FPKM (fragments per kilobase of transcript per million mapped reads) and to perform differential expression analysis [57]. Genes were defined as being expressed if they were assigned to test status “OK” (i.e., test successful) in the Cufflinks program. The false discovery rate (FDR) adjusted p-value (q value) was calculated automatically in Cuffdiff program. Genes were considered to be differentially expressed at an FDR q value < 0.05. Although the used Cufflinks pipeline included the reference-guided assembly stage, only known annotations were used in the following analysis. The RNA-Seq data were deposited in the NCBI Short Read Archive database with Accession number: PRJNA299102.

### Functional annotation

The functional analysis of DEGs (see Additional file 1) was performed using the DAVID (The Database for Annotation, Visualization and Integrated Discovery) tool (http://david.abcc.ncifcrf.gov/) [58, 59]. The *Rattus norvegicus* genome was used as the background list for the over-representation analysis in DAVID. The GO option was utilized, and the significantly (*p* < 0.05) enriched biological processes and groups of genes possibly contributing to hypertensive phenotype in ISIAH rats were identified. The Kyoto Encyclopedia of Genes and Genomes Pathway Database (KEGG, http://www.genome.jp/kegg/) was used to identify pathways that were most significant to the data set. The genes related to hypertension and CNS diseases were detected according to the DEGs annotation in Rat Genome Database (RGD, http://rgd.mcw.edu/). The detection of transcription factors among DEGs was performed using gene annotations from GenBank (http://www.ncbi.nlm.nih.gov/gene/), an atlas of combinatorial transcriptional regulation in mouse and man [60] and the Panther classification system (http://www.pantherdb.org/).

### Quantitative real-time PCR (qPCR)

The relative amount of target mRNA was measured by qPCR. Hypothalami were analyzed in 3-month old ISIAH and WAG rats (13 animals in each group). Total RNA was extracted using the TRI reagent (Molecular research center, USA). Remaining traces of genomic DNA were removed from the RNA samples using DNase I (Promega, USA) treatment, according to the manufacturer’s instructions.

cDNA was synthesized using 3 μg of total RNA, 0.25 nmol of random nonanucleotide primers (Biosan, Russia), 36 μl of reverse transcription buffer, 40 units of MoMLV (Vektor-Best, RF), and 0.4 mM dNTP. The cDNA was synthesized at 37 °C (1 h), 42 °C (30 min), and 50 °C (10 min). The enzyme was inactivated by heating the mixture at 75 °C for 5 min.

qPCR was performed in a final volume of 20 μl. The reaction volume contained a master mix with SYBR Green, forward and reverse primers (0.15 mM each), 1 unit of HotStart Taq polymerase (Vektor-Best, RF), and the cDNA template. The housekeeping gene *Rpl30* encoding ribosomal protein L30 was used as a reference gene. Primer sequences are given in Additional file 8.

qPCR was carried out in an iCycler iQ4 Real-Time PCR Detection System (Bio-Rad Laboratories, USA) with an initial denaturation of 1 min at 95 °C followed by 35 cycles of 15 s at 95 °C, 20 s at each primer’s annealing temperatures (see Additional file 8), 20 s at 72 °C, fluorescence signal acquisition for 10 s at 83 °C and generation of the melting curve from 65 °C to 94 °C. The standard-curve quantitation method was applied [61]. Standard cDNA solution for plotting calibration curves was obtained by mixing aliquots from each of the synthesized cDNA samples. In each experiment, cDNA samples with primers for the target gene (three replicates per cDNA sample), the same samples with primers for the reference gene (three replicates), and the standard cDNA dilutions (1 : 4, 1 : 8, 1 : 16, and 1 : 32) with the primers for the target gene (two replicates) and with the primers for the reference gene (two replicates) were placed on the same plate. The relative amount of the tested cDNA was determined using calibration curves derived from the dilutions of the standard cDNA. Calibration curves were built using iCycler iQ4 Real-Time PCR Detection System software. The value for the target gene was further normalized against the qPCR level of the reference gene. The relative mRNA abundance was calculated as a ratio of the normalized (mRNA/Rpl30) mRNA level in the experimental ISIAH samples to the normalized (mRNA/Rpl30) mRNA level in the control WAG samples. The normalized mRNA level in the control samples of the WAG rats was assigned a value of 1. The data points that did not fit a normal distribution (assessed by 3-sigma limits) were removed from the analysis.

### Statistical methods

Statistical calculations for qPCR data were performed with the software package Statistica v.6.0 (Statsoft, USA) using Student’s *t*-test. Differences were considered statistically significant at *p* < 0.05. The data were presented as the means and their standard errors (M ± S.E.M.).

The acquired RNA-seq data (FPKM values) were log transformed, centered and normalized. The principal coordinates method based on Euclidean metric distances was used for scaling the data sets. Then, the partial-least squares discriminant analysis (PLS-DA) approach was used to explore the pattern of co-variation for linear combinations between two blocks of variables [62, 63]. In the current experiment, the PLS-DA method and Pearson correlation were used to find a set of variables (expressed genes) that maximize the covariance between gene expression in two strains and fixed matrix with contrast values 1 and −1 for ISIAH rats and WAG rats, correspondingly. These procedures helped to construct the PLS-DA Axes maximizing the distances between ISIAH and WAG rats and to define the correlation between gene expression and PLS-DA Axis 1. The genes showing the most deviation along the first functionally meaningful synthetic PLS-DA Axis were considered to be genes that contribute the most to inter-strain differences.

### Availability of supporting data

The data sets supporting the results of this article are included within the article and its additional files.

## Additional files

Additional file 1:
**Genes differentially expressed in hypothalamus of hypertensive ISIAH and normotensive control WAG rats.** (XLS 40 kb) Additional file 2:
**Differentially expressed genes related to Gene Ontlogy (GO) terms for biological processes.** (XLS 58 kb) Additional file 3:
**Metabolic pathways and genes differentially expressed in ISIAH and WAG hypothalami.** (DOC 86 kb) Additional file 4:
**PLS-DA Axis maximizing the distance between ISIAH and WAG rats.** (JPG 57 kb) Additional file 5:
**The distribution of expressed genes along the PLS-DA Axis 1.** (JPG 117 kb) Additional file 6:
**Comparison of gene expression level measurements obtain by RNA-seq and qPCR.** (JPG 62 kb) Additional file 7:
**The summary statistics for the sequenced libraries.** (XLS 20 kb) Additional file 8:
**Primers used in real-time PCR.** (DOC 35 kb)

## Abbreviations

AA: arachidonic acid
BP: blood pressure
CNS: central nervous system
DAVID: Database for Annotation, Visualization and Integrated Discovery
DEG: differentially expressed genes
EETs: epoxyeicosatrienoic acids
FPKM: fragments per kilobase of transcript per million mapped reads
GO: Gene Ontology
HPA: hypothalamic-pituitary-adrenal
ISIAH: Inherited Stress-Induced Arterial Hypertension
KEGG: Kyoto Encyclopedia of Genes and Genomes Pathway Database
MHC: major histocompatibility complex
NK: natural killer
PLS-DA: partial-least squares discriminant analysis
qPCR: quantitative real time polymerase chain reaction
RA: retinoic acid
RGD: Rat Genome Database
RNA-seq: RNA sequencing
sEH: soluble epoxide hydrolase
WAG: Wistar Albino Glaxo
