# Supplementary material for: Genome-wide transcriptome analysis of hypothalamus in rats with inherited stress-induced arterial hypertension
Source: BMC Genet. 2016 Jan 27;17(Suppl 1):13. doi: 10.1186/s12863-015-0307-8 (PMC4895259; doi:10.1186/s12863-015-0307-8)
Supplement: Additional file 3: — Metabolic pathways and genes differentially expressed in ISIAH and WAG hypothalami. (DOC 86 kb) [file 12863_2015_307_MOESM3_ESM.doc]

Additional file 3. Metabolic pathways and genes differentially expressed in ISIAH and WAG hypothalami

| Gene symbol | Acc.# | Gene name | log2 fold_change  ISIAH/WAG |
| --- | --- | --- | --- |
| [**Autoimmune thyroid diseases**](http://david.abcc.ncifcrf.gov/kegg.jsp?path=rno04610$Complement and coagulation cascades&termId=470065447&source=kegg) **(p = 4.00E-08)** | | | |
| *Cga* | NM_053918 | glycoprotein hormones, alpha subunit | 1.15 |
| *RT1-A1* | NM_001008827 | RT1 class Ia, locus A1 | 1.17 |
| *RT1-A2* | NM_001008829 | RT1 class Ia, locus A2 | -1.98 |
| *RT1-Ba ** ^#^ | NM_001008831 | RT1 class II, locus Ba | -0.80 |
| *RT1-Bb ** ^#^ | NM_001004084 | similar to RT1 class II histocompatibility antigen, B-1 beta chain precursor | -1.26 |
| *RT1-Da* ^#^ | NM_001008847 | histocompatibility 2, class II antigen E alpha | -0.63 |
| *RT1-Db1** ^#^ | NM_001008884 | RT1 class II, locus Db1 | -0.76 |
| *RT1-M3-1* | NM_022921 | histocompatibility 2, M region locus 3 | 1.00 |
| *RT1-S3* | NM_001008886 | histocompatibility 2, T region locus 23; histocompatibility 2, T region locus 24 | -2.03 |
| *Tshb* | NM_013116 | thyroid stimulating hormone, beta | 2.05 |
| **Antigen processing and presentation (p = 7.30E-07)** | | | |
| *Cd74* | NM_013069 | Cd74 molecule, major histocompatibility complex, class II invariant chain | -0.82 |
| *Ctss* ^#^ | NM_017320 | cathepsin S | -0.50 |
| *RT1-A1* | NM_001008827 | RT1 class Ia, locus A1 | 1.17 |
| *RT1-A2* | NM_001008829 | RT1 class Ia, locus A2 | -1.98 |
| *RT1-Ba ** ^#^ | NM_001008831 | RT1 class II, locus Ba | -0.80 |
| *RT1-Bb ** ^#^ | NM_001004084 | similar to RT1 class II histocompatibility antigen, B-1 beta chain precursor | -1.26 |
| *RT1-Da* ^#^ | NM_001008847 | histocompatibility 2, class II antigen E alpha | -0.63 |
| *RT1-Db1** ^#^ | NM_001008884 | RT1 class II, locus Db1 | -0.76 |
| *RT1-M3-1* | NM_022921 | histocompatibility 2, M region locus 3 | 1.00 |
| *RT1-S3* | NM_001008886 | histocompatibility 2, T region locus 23; histocompatibility 2, T region locus 24 | -2.03 |
| **Type I diabetes mellitus (p = 1.20E-05)** | | | |
| *RT1-A1* | NM_001008827 | RT1 class Ia, locus A1 | 1.17 |
| *RT1-A2* | NM_001008829 | RT1 class Ia, locus A2 | -1.98 |
| *RT1-Ba ** ^#^ | NM_001008831 | RT1 class II, locus Ba | -0.80 |
| *RT1-Bb** ^#^ | NM_001004084 | similar to RT1 class II histocompatibility antigen, B-1 beta chain precursor | -1.26 |
| *RT1-Da* ^#^ | NM_001008847 | histocompatibility 2, class II antigen E alpha | -0.63 |
| *RT1-Db1** ^#^ | NM_001008884 | RT1 class II, locus Db1 | -0.76 |
| *RT1-M3-1* | NM_022921 | histocompatibility 2, M region locus 3 | 1.00 |
| *RT1-S3* | NM_001008886 | histocompatibility 2, T region locus 23; histocompatibility 2, T region locus 24 | -2.03 |
| **Cell adhesion molecules (CAMs) (p = 2.70E-04)** | | | |
| *RT1-A1* | NM_001008827 | RT1 class Ia, locus A1 | 1.17 |
| *RT1-A2* | NM_001008829 | RT1 class Ia, locus A2 | -1.98 |
| *RT1-Ba ** ^#^ | NM_001008831 | RT1 class II, locus Ba | -0.80 |
| *RT1-Bb** ^#^ | NM_001004084 | similar to RT1 class II histocompatibility antigen, B-1 beta chain precursor | -1.26 |
| *RT1-Da* ^#^ | NM_001008847 | histocompatibility 2, class II antigen E alpha | -0.63 |
| *RT1-Db1** ^#^ | NM_001008884 | RT1 class II, locus Db1 | -0.76 |
| *RT1-M3-1* | NM_022921 | histocompatibility 2, M region locus 3 | 1.00 |
| *RT1-S3* | NM_001008886 | histocompatibility 2, T region locus 23; histocompatibility 2, T region locus 24 | -2.03 |
| *Selplg* ^#^ | NM_001013230 | selectin P ligand | -0.78 |
| **Retinol metabolism (p = 1.50E-02)** | | | |
| *Aldh1a2* | NM_053896 | aldehyde dehydrogenase 1 family | -0.69 |
| *Cyp4x1* | NM_145675 | cytochrome P450, family 4, subfamily x, polypeptide 1 | -2.01 |
| *Cyp26b1* | NM_181087 | cytochrome P450, family 26, subfamily b, polypeptide 1 | -1.11 |
| *Retsat* | NM_145084 | retinol saturase (all trans retinol 13,14 reductase) | 1.82 |
| **Arachidonic acid metabolism (p = 2.30E-02)** | | | |
| *Cyp2j10* | NM_001134980 | cytochrome P450, family 2, subfamily j, polypeptide 10 | -0.94 |
| *Cyp4x1* | NM_145675 | cytochrome P450, family 4, subfamily x, polypeptide 1 | -2.01 |
| *Ephx2** ^#^ | NM_022936 | epoxide hydrolase 2 | 4.40 |
| *Ptgds** ^#^ | NM_013015 | prostaglandin D2 synthase | -0.57 |

ISIAH and WAG – rat strains used in the study. Genes associated with: **-* hypertension; ^#^- central nervous system diseases;
