# Supplementary figures and images for: Genome-wide transcriptome analysis of hypothalamus in rats with inherited stress-induced arterial hypertension
Source: BMC Genet. 2016 Jan 27;17(Suppl 1):13. doi: 10.1186/s12863-015-0307-8 (PMC4895259; doi:10.1186/s12863-015-0307-8)

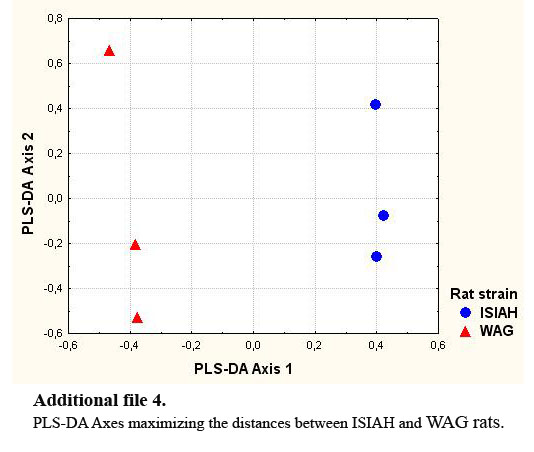

Supplement: Additional file 4: — PLS-DA Axis maximizing the distance between ISIAH and WAG rats. (JPG 57 kb) [file 12863_2015_307_MOESM4_ESM.jpg]

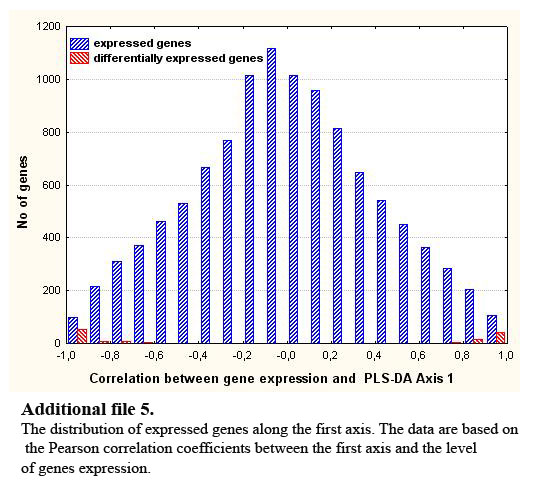

Supplement: Additional file 5: — The distribution of expressed genes along the PLS-DA Axis 1. (JPG 117 kb) [file 12863_2015_307_MOESM5_ESM.jpg]

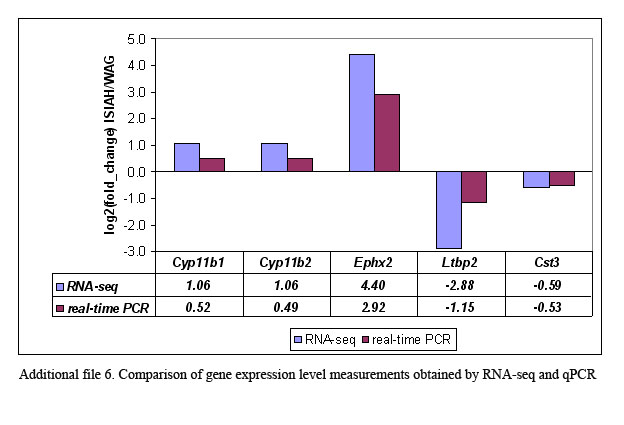

Supplement: Additional file 6: — Comparison of gene expression level measurements obtain by RNA-seq and qPCR. (JPG 62 kb) [file 12863_2015_307_MOESM6_ESM.jpg]
