# Supplementary material for: Genome-wide transcriptome analysis of hypothalamus in rats with inherited stress-induced arterial hypertension
Source: BMC Genet. 2016 Jan 27;17(Suppl 1):13. doi: 10.1186/s12863-015-0307-8 (PMC4895259; doi:10.1186/s12863-015-0307-8)
Supplement: Additional file 8: — Primers used in real-time PCR. (DOC 35 kb) [file 12863_2015_307_MOESM8_ESM.doc]

**Additional file 8.**

Primers used in real-time PCR

| Gene | Primers, 5’--->3’ | | Length of PCR fragment, bp | Annealing temperature, ^o^C |
| --- | --- | --- | --- | --- |
|  | forward | reverse |  |  |
| *Cyp11b1* | CATGGAAGCCAGCCATTTTGT | GTGAATGTCACGCTCTCAGGT | 84 | 62 |
| *Cyp1b2* | TGTGGCAGCACTAATAACTCA | GCCAGCTCAAAAAGGGTCA | 126 | 62 |
| *Cst3* | CCAATGGTCCCTTACTTGTTC | GCAGCTTCTTTACTGTCTCC | 114 | 62 |
| *Ephx2* | TTTCTTGGAGGTACCAG↓ATCC | CAGTCATGGCCAATGAACAC | 193 | 64 |
| *Ltbp2* | AGCCTCGCCTCCTTTTTATC | AGCGACTTCGTGTTTTGTCT | 202 | 62 |
| *Rpl30* | ATGGTGGCTGCAAAGAAGAC | CAAAGCTGGACAGTTGTTGG | 143 | 62 |
